# Psychological Antecedents of Retirement Planning: A Systematic Review

**DOI:** 10.3389/fpsyg.2018.01870

**Published:** 2018-10-09

**Authors:** Matthew J. Kerry

**Affiliations:** Department of Management, Technology, and Economics, The Swiss Federal Institute of Technology (ETH-Zürich), Zurich, Switzerland

**Keywords:** retirement plan, planning, preparation, saving, individual differences (IDs)

## Abstract

As workforce aging continues through the next decade, the number of persons who will retire from long-held jobs and careers will increase. In recent years, researchers across disciplines of psychology have focused attention on the impact of the retirement process on post-retirement adjustment and well-being. The objective of the current review is twofold. The first goal is to review the literature on retirement planning with attention to past conceptualizations and current theoretical specifications. Second, empirical work investigating the psychological antecedents of retirement planning is reviewed. The primary conclusion reached from this review is that, conceptually, retirement planning continues to be poorly delineated and, thereby, narrowly investigated. Empirically, cognitive antecedents of retirement planning continue to figure prominently in both workplace and retirement researches. The boundary conditions of retirement planning, as well as alternative mechanisms for adult wellbeing, are discussed. Specifically, retirement planning's meaning amidst increasing job mobility and longer life expectancies are identified as two complementary areas for future empirical integration of work–retirement research domains.

Career concerns will make the agent averse to ambiguity if he is already averse to risk.

—Eric Rasmusen, Career Concerns and Ambiguity Aversion

Industrial/organizational (I/O) psychology's research on retirement, to date, has focused on the changing nature of retirement and the implications of this event for older workers and retirees (Ekerdt, 2010; Wang and Schultz, 2010; Feldman and Beehr, 2011; see reviews by, Adams and Rau, 2011; Wang and Shi, 2014). Organizational scholars have also recognized the parallel-changes in the nature of retirement and that of work (Shultz et al., 2013). For example, the increasingly complex conditions surrounding retirement may be reflected in organizations' increasing emphasis on work flexibility; the common feature being work-life balance, and the common function being employee attraction-retention, respectively (Unger et al., 2015). Consider, for illustrative purpose, how the holistic continuity of phased-workforce withdrawal complements the concurrency of work-recovery cycles (Zijlstra and Sonnentag, 2006).

Retirement researchers have chiefly examined one direction of this link, that is, how the work-to-retirement transitions affect retiree outcomes (c.f., Ekerdt, 2004). Far less attention has been paid to the topic of how normative changes associated with the retirement process influence younger cohorts' approach to employment and retirement planning. Cascio's (1995) seminal review on the “redefinition of work” might evidence this oversight, where career retirement and workforce aging are both unaddressed. This discrepancy is substantive, inasmuch as it implicates employees' career aspirations and preparations for retirement, which can be expected to impact, not only their ongoing worklife decisions but also, their future post-retirement wellbeing. To further illustrate this discrepancy, we plot the relative-growth rates of publications on retirement planning (RePlanning) by antecedents and outcomes in Figure 1 below.

**Figure 1:**
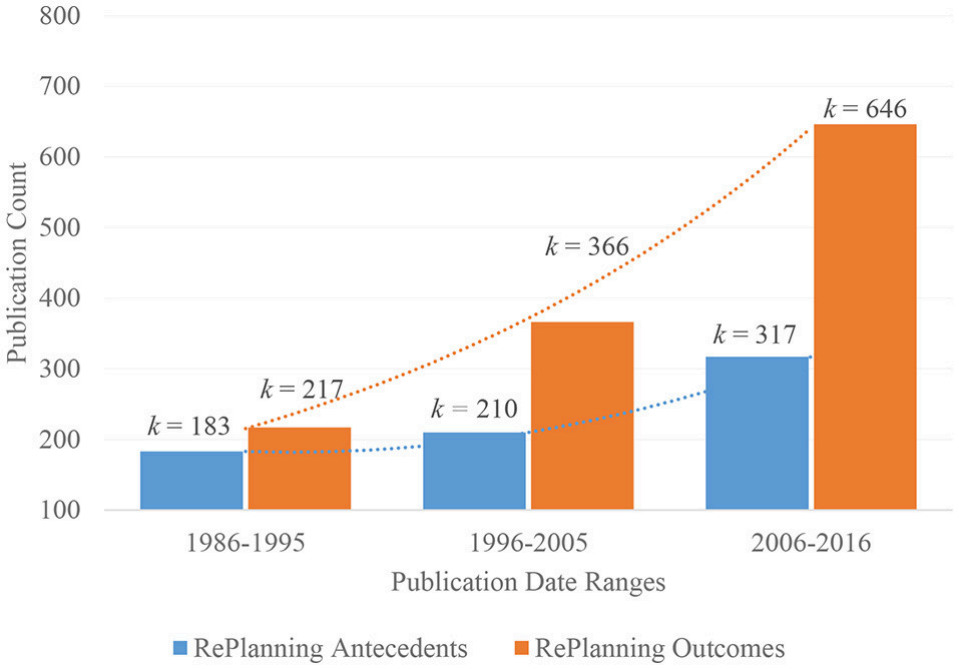
Summary “retirement planning” publication-rate since Beehr (1986) seminal article, separated by “antecedents” and “outcomes”.

The purpose of the current is to review and index the empirical evidence on individual-antecedents of RePlanning for continued age-integration of work and social institutions. First, we elaborate on existing research paradigms of RePlanning below. After contextualizing RePlanning as our focal construct, the next section will explicate primary conceptualizations. After this Introduction, we review the empirical evidence on psychological antecedents of RePlanning.

As a focal construct, RePlanning inherits many of the complexities surrounding the “famously ambiguous” criteria of retirement itself (Ekerdt, 2010; p.70). That is, from a normative perspective there is no longer an abrupt end to one's work life, but rather, pluralistic pathways exist that one can take in the transition from full-time employment to full-time retirement (Adams and Beehr, 2003). Epistemologically, psychologists have organized retirement research into three non-exclusive paradigms: (1) decision-making (DM), (2) transition adjustment, and (3) career development (see Wang and Schultz, 2010). Conceptually, *RePlanning* is typically located between the DM and transition adjustment perspectives (Wang and Schultz, 2010). That is, RePlanning not only influences the decision to withdraw from the workforce, but it also has consequences for subsequent adjustment and well-being.

Phenomenological perspectives of RePlanning are also characterized by process-models (see Gall et al., 1997; Taylor and Doverspike, 2003). While the decision to retire has never been conceptualized as a discrete act (Beehr, 1986; Feldman, 1994), some retirement scholars have suggested that individuals anticipate and develop retirement intentions from their initial point of entry into the workforce (Ekerdt, 2004). In their review of the psychological paradigms of retirement research, Wang and Schultz, 2010 recognized a disjunction between the historical DM and contemporary retirement perspectives, stating, “…few studies that examined outcomes of retirement have incorporated factors that influenced the original retirement decision…This creates a logic gap because the reasons *why* people decide to retire would naturally influence *how* they evaluate outcomes associated with their retirement.” (p.176)^1^. Interestingly, similar antecedent-outcome gaps were earlier observed *within* the DM paradigm by Ekerdt et al. (1996).

Given the analogous claims above, spanning a quarter-century of retirement research, a topical substantiation of the antecedent-outcome gap seemed warranted. As an instructive example, we scored empirical studies of bridge-employment (BE) for inclusive-assessment of employees' “plans” or “intentions” for retirement. From this Table 1, claims for antecedent-outcome gaps were corroborated, although we may cautiously conclude, at least in the exemplar case of BE, that the antecedent-outcome gap is narrowing.

**Table 1:** Summary bridge-employment studies scored by retirement “planning” or “intentions” inclusion.

**Study**	**Outcome**	**Plane**	**Intent**
Kim and Feldman, 2000	Amount of bridge employment; University- vs. non-university BE.	✓ ret. counsel; *r* = −0.01–0.03	x
Wang et al., 2008	Career BE vs. non-career BE vs. full-retirement	✓ ret. thoughts; *r* = −0.06–0.16^*^	x
Von Bonsdorff et al., 2009	Career BE vs. non-career BE vs. retirement *intentions*	x	–
Gobeski and Beehr, 2009	Career vs. non-career BE	x	x
Zhan et al., 2009	Disease, functional, and mental health	x	x
Jones and McIntosh, 2010	Turnover *intention*, organizational-BE, career-BE, non-career BE, retirement	x	–
Pengcharoen and Shultz, 2010	Continued work vs. BE vs. Full-retirement	✓ friend discus; _n.s._ spouse discus; +	x
Zaniboni et al., 2010	Full-retirement vs. part-retirement vs. job mobility *intentions*	x	–
Mariappanadar, 2013	Contingent- vs. flexible-type BE	✓ financial prep; *r* = 0.20^*^, −0.24^*^	x
Wang and Shi, 2014	Career vs. non-career BE; Organization vs. non-organization BE	x	x
Dingemans and Henkens, 2014	Failed BE (*intended*) vs. successful BE Life satisfaction	x	✓ failed BE; *r* = −0.44
Kalokerinos et al., 2015b	BE interest	✓ nearer ret; *r* = −0.08	✓ no reentry; *r* = −0.39^*^
Zhan et al., 2015	BE participation	x	✓ finance; *r* = 0.27^*^

* *p < 0.05, n.s, non-significant*;

x *, not included*;

✓ *= included*.

+ *= unspecified estimate. BE, bridge-employment. Entries listed in published-chronological order. Ret, retirement*.

As an earlier solution, Ekerdt et al. (1996) drew on Azjen and Fishbein's (1980) theory of planned behavior (TPB) to proposed retirement intentions, operationalized as self-reported plans, as a viable alternative for abridging retirement's evaluative-gap. Specifically, Ekerdt et al. (1996) developed a five-category instrument designed to capture older (51–61-years) employees' “current ideas about next or proximate transitions, not their notions about patterns of possible moves or the final situation” (p. 141). Using baseline data from the U.S. Health and Retirement Study (HRS), Ekerdt et al. (1996) reported preliminary evidence for the usefulness of their heuristic taxonomy of retirement plans. Mapping these five “plan categories” from Ekerdt et al. (1996) onto the newer, outgrowth paradigms of retirement research helps to illustrate the value-added aspect of the DM perspective (see Figure 2). Specifically, the first three planning categories have a direct relation with newer retirement-research paradigms. More interesting, however, was the finding that roughly half of respondents reported having, either “no plans” for retirement, or “planned not to” retire. In a sample of 51–61 year olds, this substantive proportion of the sample would, at best, have only indirect relations (hashed arrows) with the newer “transition/adjustment” perspective.

**Figure 2:**
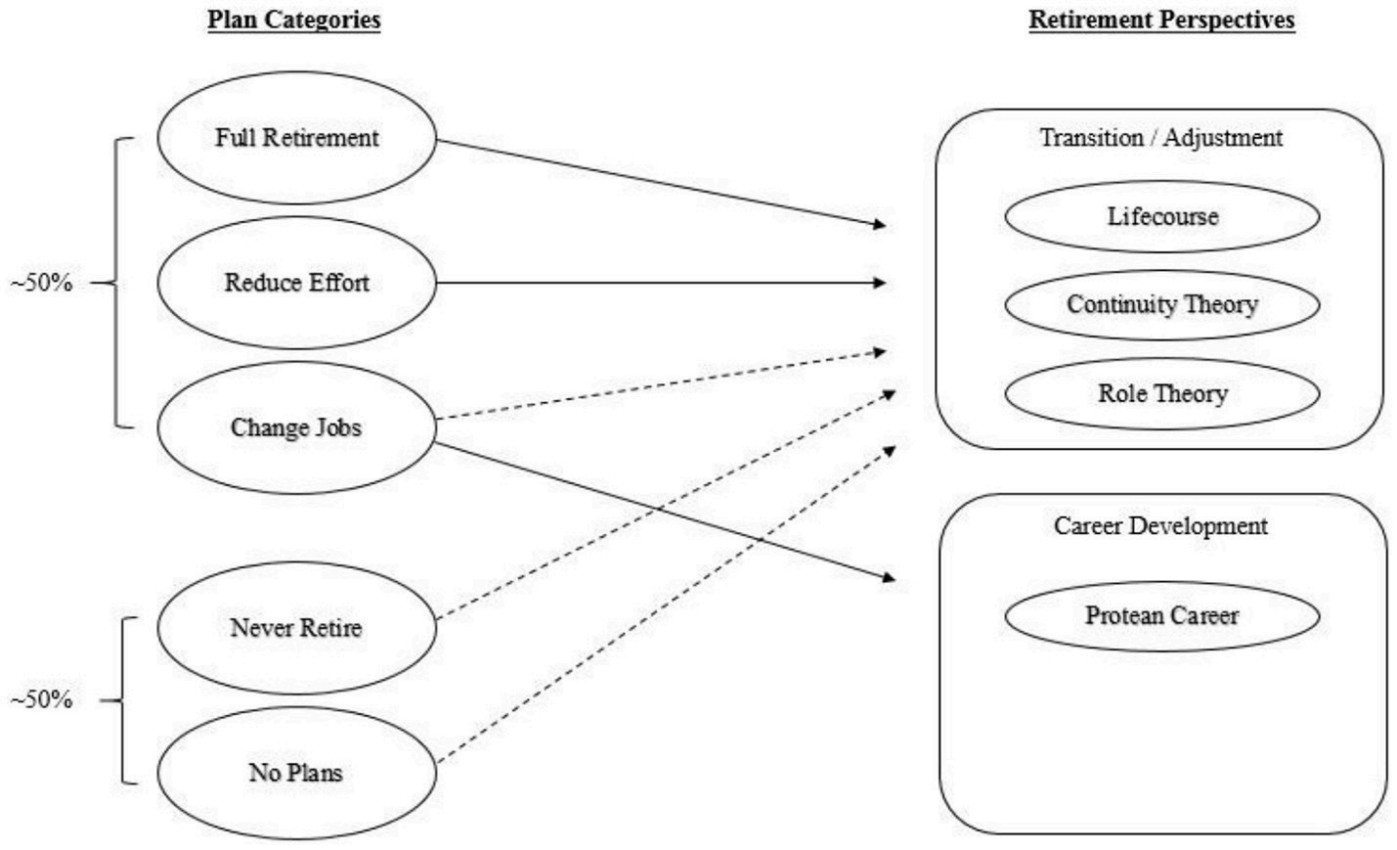
Relative prevalence of plan categories from first-wave Juster (1992) and their conceptual relations with retirement's psychological perspectives.

Given the positive outcomes associated with RePlanning for both retirees and employees, it may seem perplexing that the largest proportion of individuals reported having “no plans” whatsoever (~ 40%). On the basis of Wang and Schultz (2010) logic regarding the nature of retirement decisions (RDs) influencing outcome evaluations, it is tenable that states of *indecision* have implications for perceived outcomes. In fact, Ekerdt et al. (2001) has advocated for furthering research on those with *no plans* (i.e., a state of retirement uncertainty) as a psychologically meaningful stance toward retirement. For example, arguably, phased-retirement paths via non-occupational bridge-employment (vocational sampling) could substitute for long-term planning. Assessing specific plans (intentions) for the post-employment period should prove to be the best predictor of future behavior. Viewed as a “behavioral family,” these forms of retirement necessarily have commensurately complex antecedents (Hanisch, 1995), which have not yet been accounted for by extant research.

In the next section, we narrow our scope on psychological conceptualizations of RePlanning. A critical-positivist examination of the empirical evidence on RePlanning's antecedents follows.

## Historical conceptualizations and operationalizations of retirement planning

Historically, retirement planning has been delineated by formality, based on a distinction between employer offerings (formal) and employee-directed activities (informal). More recent, planning content has become the focus of psychological research, with the non-financial category being expanded to include, for example, health, psychosocial, and leisure domains of planning activities. After chronicling the process- and content-based conceptions of RePlanning, this section concludes with a brief discussion on potentially meaningful separation of planning and preparation in work-future retirement research.

### Planning process—formal vs. informal

Notably, descriptive models from behavioral economics (Kahneman and Tversky, 1979, 2013; planning fallacy) and predictive models from psychology (Azjen, 1991) do not delineate planning behavior in terms of its degree of *formality*. However, accepting RePlanning as a contextual construct (Coan, 1964), the earliest distinction regarding formality can be sourced to Kroeger (1982). Specifically, Kroeger ascribes *formal planning* and *informal planning* to employer-sponsored and employee-directed activities, respectively. Beehr (1986) also refers to formal and informal RePlanning, though only formal planning is implied as constituting employer-sponsored programs, consistent with Kroeger (1982).

Using interview data from recent-retirees (< 2 years), Kroeger reported evidence for gender-differences in the types of RePlanning conducted, with men more likely to engage in informal planning compared to women. Evidence was also reported for the direct effect of retirement anticipation on the planning process, with greater anticipation of the work-retirement transition accentuating observed-gender differences. Taylor-Carter et al. (1997) characterization of formal RePlanning followed precedent, viewing it as participation in an employer-sponsored workplace event. Informal planning, however, was temporally broadened by defining it as an employee's tendency to acquire retirement information *over the course of one's career*. This seems to be an important distinction because, temporally, the formality–informality distinction could be viewed analogously to episodic–continuous planning. Another contribution of Taylor-Carter et al. (1997), is their *functional* specification of formal and informal planning. Specifically, whereas formal planning interventions only impacted self-efficacy, whereas informal planning impacted both self-efficacy and expected satisfaction in retirement.

Citing proliferation of imprecise RePlanning measures, Noone et al. (2010) constructed a multi-domain, planning-stage process self-report. Defining *RePlanning* as “…goal-directed thoughts and behaviors that promote good health and provide financial security, fulfilling lifestyles, and rewarding roles in retirement” (p. 521), the authors essentially equate *informal* and *non-financial* planning. The conflation accords with the authors' subsequent claim for constructing a new RePlanning questionnaire, “…financial planning can be divided into two broad categories: pension, savings, or retirement wealth and more informal modes, such as financial advice seeking and the consumption of educational material. The measurement of the former…is not considered in detail here” (p.521). This conclusion implies the incomplete measurement of financial retirement planning domains. Retirement planning's domain specificity is considered in greater detail in the next section below.

### Planning domain—financial vs. non-financial

Preliminary discriminant-validity evidence for RePlanning domain-specificity was reported by MacEwan et al. (1995). In a prospective cross-sectional design (*N* = 217), financial planning was found to predict expected-financial satisfaction, whereas activity planning predicted both expected-financial satisfaction and expected-well-being in retirement.

Over the past two decades, Hershey and colleagues have advanced a substantive program of psychological research on RePlanning. While never formally distinguishing between financial and nonfinancial domains, the conceptualization and measurement approach stemming from this program has been informative. In their first, prospective cross-sectional design, Hershey and Mowen (2000) examined the nomological net of various affective and conative ID variables with *financial preparedness*—defined as competency for retirement's financial demands associated with retirement. The authors similarly conceptualized *financial planning* as self-rated knowledge of the planning process (a scale that is retained in subsequent studies). In addition, the authors adapted a measure of retirement *involvement*, utilizing a semantic differential response format. Results of an exploratory factor analysis indicated two separable dimensions: (1) “retirement affect reactions,” and (2) “retirement relevance.” Correlational findings indicated that “financial planning” was closer associated with financial preparedness (*r* = 0.57), compared to retirement “affect” (*r* = 0.30) or “relevance” (*r* = 0.17).

Hershey and Mowen's (2000) pattern of findings was subsequently extended in a set of three studies. First, in another cross-sectional design, the finding was extended to a single-item indicator of retirement savings behavior (*r* = 0.51) (Jacobs-Lawson and Hershey, 2005). In a second study, relatively smaller effects were reported (*r* = 0.19) when examining “financial planning” correlated with a multi-item behavioral frequency scale. The scale was designed to assess *RePlanning activities*, defined as the “frequency of both information seeking and instrumental planning activities” (p. 31; *r* = 0.19). In a third study, information-seeking and instrumental planning was further explicated as, increasing declarative knowledge and calibrating retirement expectancies, respectively (Stawski et al., 2007). Despite receiving empirical evidence for factorial-distinction, the authors conducted analyses with the composite activities instrument, reporting a medium-sized effect with self-reported savings (*r* = 0.38).

Unique to the series of studies reviewed above, Muratore and Earl (2010) formally defined *retirement preparation*, as “…effort invested by individuals, while still employed, to provide for their wellbeing in retirement” (p.99). The subjective effort-based measure was conceptualized as a proxy for implementation intentions (see Gollwitzer, 1999). Three functional domains delineate financial and non-financial content as follows: (1) *public protection*—strategies involving both financial and nonfinancial benefits supplied by government (e.g., public pensions, public health benefits, housing programs), (2) *self-insurance*—financial strategies for optimizing retirement wealth (e.g., savings, personal investment in private insurance or annuities), and (3) *self-protection*—non-financial strategies for optimizing retirement wellbeing (healthful lifestyle, social engagements). Findings indicated, that age exhibited positive correlates across financial categories (*r* = 0.17, 31), whereas income exhibited differential correlates across the same categories (*r* = −0.26, 0.17). Both demographic variables exhibited negligible correlates with the *non-financial* category (*r* = 0.06. −0.02, respectively). Further, the only psychological variable included in the study, core self-evaluations, exhibited a positive correlate with the non-financial, category, *self-protection* (*r* = 0.24).

In summary, the findings for negligible demographic correlates with non-financial planning, but significant correlates with core self-evaluations, suggest the unique importance of non-financial domains in relation to psychological variables. The finding is also consistent with recent meta-analytic (MA) evidence from Topa et al. (2012), which indicated that objective income was a weaker correlate of RePlanning (*r* = 0.18) than affect-laden subjective indicators (*r* = 0.31). A single-sample *Fisher's r-z* transformation further indicated a statistically significant difference between objective-subjective indicators of RePlanning, *z*_(1)_ = −2.80, *p* < 0.01.

**Figure 3:**
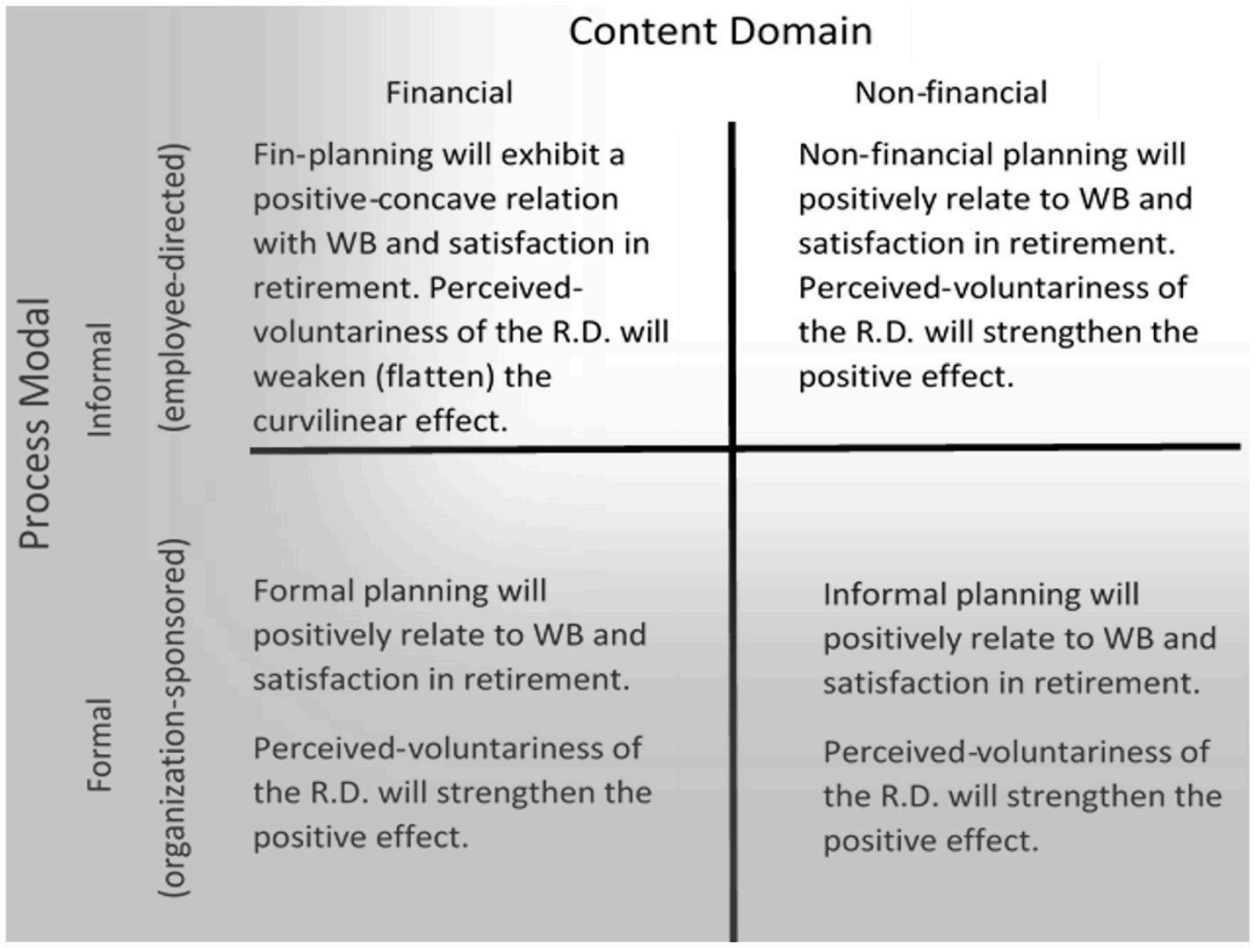
Summary-descriptive propositions for different forms of retirement planning and decision-voluntariness for retiree outcomes. The hashed-axes represent RePlanning emphasis-shift. RD, retirement decision.

To conclude this section, we submit Figure 3 below to organize descriptive-propositions relating formality-fiscality conceptions of RePlanning to outcomes. We tentatively-specify anticipated interactions with perceived-voluntariness to ground the propositions. The next section briefly elaborates on terminological specifications derived from these in/formal and non/financial historical distinctions of RePlanning. In Figure 3, shadowing helps to illustrate RePlanning's trend away from conventional paradigms' focus on formal / financial aspects to current paradigms' broadened consideration of informal / non-financial aspects of RePlanning.

### Terminological specificity—planning vs. preparation

As indicated in the previous section, historically, the conceptualization and operationalization of RePlanning has suffered from definitional shortcomings. Unsurprisingly, failure to define the focal construct in past studies has led to imprecise terminology, as well. For example, “preparation” is interchanged with “planning” to describe employees' retirement-related activities. There are a number of points to consider before the *preparation-planning* distinction is presumed inconsequent. For scope, only a few such points are elaborated herein.

Early gerontology theory articulated the ideographic pathways to retirement and defined *preparation* as one's reaction to past experiences and the early cultivation of interests and hobbies, *i.e., avocations* (Moore, 1946). Interestingly, at lifespan's disciplinary counterpoint, educational psychologist Darrel H. Hart may be credited for formally distinguishing *preparation-planning* in career counseling, specifically, for occupational *entry* (1970). According to Hart, Rayner, and Christensen's classificatory model, “…planning is defined as actively seeking a job, and preparation referred to past experience, academic or on the job training” (1971; p.280). Also included in Hart and colleagues' model was, “unplanned, situational events,” termed *chance* events (1971; p.280). This occupational-theory break from strict-rational models has seeded many contemporary, career-development concepts, e.g., *protean career*—Hall (1996); *planned happenstance*—Mitchell et al. (1999); *job crafting*—Wrzesniewski and Dutton (2001). The circulation of *preparation*, from occupational-entry to retirement-as-career development implicates contexts of justification for further theorizing on preparation–planning distinctions (Kuhn, 1970).

Abiding theoretical development, distinguishing preparation-planning holds practical implications for organizations, employees, and retirees. For organizations, a provocative situation to consider is the indirect transaction-cost incurred by older employees when workplace conditions deteriorate (*e.g., ageism*) or economic markets contract (pension reduction). For example, under sufficient preparation and no intention to retire, an older employee could voluntarily turnover to a less-discriminatory organization or exhibit costlier presenteeism for fear of dismissal, respectively (Benjamin et al., 2009). Alternatively, under insufficient preparation and intention to retire, an older employee may reduce (increase) citizenship (deviant) workplace behaviors or experience *job-lock*, a complement to embeddedness that has received scant research, c.f., Fisher et al. (2016). Benjamin et al. (2009) reported findings, however, that indicate older workers' job-lock is positively related to *presenteeism* (*r* = 0.18). It is be tenable that, some localized (workplace) episodes of presenteeism are symptomatic of more general, perhaps “deliberate” job-lock (e.g., Johns, 2010). Abiding corollaries between organizational costs of presenteeism and age-related infirmities (e.g., productivity, contagion, and accidents), differential prediction may approximate discrepancies in one's sufficient preparation (exit-choice) and planning (withdraw-intention) for retirement.

For individuals, the planning-preparation distinction is also important, inasmuch as the normative prevalence of retirement increases. For example, increased emphasis on RePlanning presumes that everyone who enters a career intends to exit that career by transitioning into retirement. Consequently, precise estimation of retiree wellbeing from planning, if conflated with preparation, will necessarily be limited. Inversely, recent empirical evidence underscores the substantive impact of *perceived-voluntariness* of retirement on, both employee anxiety (van Solinge and Henkens, 2008) and retiree well-being (Hershey and Henkens, 2013). Put simply, the positive effects inferred by retirement plans on retiree well-being should reasonably be limited by plan fulfillment. Evidence indirectly supporting this supposition comes from longitudinal data indicating greater life dissatisfaction over unemployment periods for individuals higher in trait-conscientiousness (Boyce et al., 2010). To unintended consequences of retirement, Ekerdt (2004) summarizes,

“Retirement may become de-standardized as to the incidentals of timing and form, but not as to its eventuality…There is some irony in a stronger retirement norm. Retirement saving is being urged upon the public so that individuals will have wealth that should eventually give them options, choices, and control in decisions about when and whether to work. Instead, the saving effort and the emphasis on it solidifies the desire for, demand for, and norm of retirement, thus creating a constraint on behavior.” (p. 6).

The counterfactual of plan fulfillment is more disconcerting. That is, workers lacking defined careers (or sufficient RePlanning) should not imply indifference or disinterest toward retirement. To wit, if greater life expectancies were temporally commensurate with normative retirees, public mandates for retirement (as for occupational entry, for that matter) would be obsolete. First-time experimental evidence for dysfunction of insufficient RePlanning has recently been published where, individuals randomized to a shorter life expectancy indicated intentions for later retirement if they reported insufficient RePlanning (Kerry and Embretson, 2017).

### Summary of historical conceptualization and operationalization

Consolidated in Online Appendix A, heterogeneity characterizes the conceptualizations and assessments of RePlanning. A few points are notable with emphasis for future empirical research. First, single-item indicators may be particularly problematic for precise estimation in cohort-studies due to correlated errors. Second, varietal items and response-category formats can be expected to introduce scaling artifacts that make comparability of findings difficult. For these two measurement model issues, we recommend adoption of advanced psychometric methods in future RePlanning research (see Embretson and Reise, 2000). Third, regarding sampling models, population representativeness should be prioritized when selecting (excluding) initial item pools in RePlanning instrument development. For example, two multidimensional instruments currently administered in nationally representative surveys were piloted on narrower, single-source samples (academic faculty, Noone et al., 2010; financial firm employees; Petkoska and Earl, 2009).

A brief exposé on terminology indicated theoretical and practical misspecifications from interchanging *planning* with *preparation* in extant research. Practically, planning's distinction from preparation may be understood along lines of intentionality.

It will be important for future psychological research on RePlanning to better-articulate what does, and does not, constitute the focal construct (Dawes, 1995). The value of parsing planning from preparation should be empirically determined and interpreted within-context. For example, for organization retention, understanding the incremental impact of plan fulfillment over financial preparation may bear on RePlanning's psychological utility. If retiree wellbeing is the focal outcome-of-interest, then estimates should be unconfounded by career intentions, avocational plans, and retirement voluntariness. Despite the many challenges noted above, a wealth of empirical research on the RD has been readily extended to the RePlanning domain. In the subsequent sections, the empirical evidence for antecedents linked to RePlanning are reviewed. An abbreviated section on situational influences precedes this review's primary focus on psychological (personlogic) antecedents of RePlanning. Prefacing the empirical literature findings, however, a brief methodological overview of the systematic search protocol that was used for scoping the literature to identify studies and extract empirical results is presented below.

## Brief methodological overview of replanning systematic-literature search protocol

Given the comprehensive coverage endeavored by this review across heterogeneous sampling of psychological individual differences, a systematic meta-narrative review was conducted for flexibility over more stringent/narrow synthesis from meta-analytic procedures. The methods conducted were framed in accordance to the the preferred reporting items for systematic reviews—analyses standards (PRISMA; Moher et al., 2009).

To scope and identify relevant RePlanning studies, a systematic literature search was conducted covering un- /published research dating from Beehr's (1986) seminal article to current. Details of our Boolean-keyword search protocol results is provided in Online Appendix Table B. Five scientific databases and search engines were utilized for adequate sample coverage of the social sciences literature, including ProQuest, Medline, JSTOR, ERIC, and Google Scholar. Titles and abstracts were keyword-searched twice for variants of “antecedents”/“determinants”/“predictors”/“causes” concomitant with RePlanning. Studies were primarily excluded for domain irrelevance (non-psychological variables included) or insufficient reporting (failing to report bivariate empirical associations between antecedents and RePlanning). We included studies that focused on the links between psychological factors and retirement planning. The focal population included both older workers (prospective planning) and retirees (retrospective planning). For comprehensiveness, we included all study designs (experimental and observational). After identifying a potential of *k* = 89 studies for inclusion, a subsample of independently screened (*k* = 20) Abstracts commenced and resulted in *k* = 51 total studies retained for inclusion in the current review. Empirical findings on the non-psychological influences of RePlanning is presented briefly in the next section. Non-Psychological Influences on ReTIREMENT Planning.

We must parameterize the remainder of our review for clarity-of-scope. First, we restrict our focus to the individual-level of analysis (Rousseau, 1985) (c.f., Taylor and Schaffer, 2012). Second, our individual-level is focused on studies of personlogic-traits for the main text herein (c.f., Mischel, 1968; Meyer et al., 2010). Interested readers are encouraged, however, to see our online appendix (Appendix C) summarizing demographic and situational correlates of RePlanning. Third, in principle, an epistemological perspective of RePlanning is adopted, but we note that the increasing overlap between work - retirement domains demands substantive ontological perspectives in future empirical research, as well. Below, we briefly overview a few, substantive findings from the empirical literature of demographic and situational antecedents of RePlanning.

Retirement scholars have long-recognized that comprehension of the retirement process necessitates an understanding of employees' valuation of work (Friedman and Havinghurst, 1954). Recent lifespan theories of motivation provide a reasonable lens for understanding how the non-monetary aspects of work could, tenably, deter employees from engaging in RePlanning. At the task-level (e.g., autonomy, variety), mixed-support has historically been reported as-to expected effects of job characteristics on intentions and RePlanning (Hanisch and Hulin, 1990) (c.f., Taylor and Shore, 1995; Kosloski et al., 2001). Current meta-analytic evidence from Topa et al. (2012), however, supports the general assumption, that is, job satisfaction exhibited the strongest correlate with RePlanning (*r* = −0.26). This finding provides indirect support for work-design perspectives toward organizational retention of older workers. By the same token, aversive work conditions should, *ceteris paribus*, intensify RePlanning and accelerate organizational exit (see William Lee et al., 2014). Topa's (2012) findings also corroborate this assumption, with negative work conditions exhibiting the strongest correlate with bridge employment (*r* = −0.31). The increasing empirical attention to retirement-expectancy and -voluntariness should further clarify the nomological network of work-related factors related to retirement planning (see Fisher et al., 2016).

Compared to work-related factors, demographic and biographic correlates of RePlanning are relatively more consistent. Historical gender- and race-disparities in RePlanning appear to be decreasing with emerging labor force-opportunity structures (Helman et al., 2015). Determining whether empirical gaps in RePlanning are owed to resource limitations or financial risk-aversion is an important undertaking for designing and developing future RePlanning interventions. This pertains to the epigraph used at the start of this review; That is, if increasing mobility is perceived as risk rather than opportunity, then indecision and complacency may be an unintended consequence of incumbent employees to organizations. Marital status continues to exhibit robust-positive correlates with RePlanning, which generally concords with tenets of lifespan theory, which predict greater valuation and selectivity of emotional-support networks with age.

In principle, the usefulness of chronological age as a predictor of planning behavior generally has not been well understood (Wohlwill, 1970). Empirical evidence indicating that “expected retirement age” and “perceived proximity until retirement” are better predictors of planning than chronological age, may, indirectly reflect the failure to account for retirement intentions as an important antecedent of planning. Reifications of “contextual age” are well-motivated, but likely of modest improvement toward understanding, particularly vis-à-vis a more fundamentally erroneous assumption stemming from historical-antipodes of lifespan disciplines (see Kerry and Embretson, 2017). We elaborate more on this in a later section, suffice, it is analogous to the theoretical circulation of *preparation*, from occupational entry to retirement. Similarly, we postpone further elaboration of wealth and health correlates for our discussion section.

To conclude this brief section, many of the situational variables that putatively affect the RD have exhibited similar patterns of correlates to RePlanning. Demographic indicators also continue to play an important role in elucidating the planning behavior of future retirees. To the contrary, chronological age, as a predictor of planning behavior is not well understood (Wohlwill, 1970). Studies on the topic of race and gender, are also deserving of greater research attention. Determining whether empirically-documented gaps in planning are owed to resource limitations or financial risk-aversion is an important undertaking for designing and developing interventions.

## Psychological antecedents of retirement planning

This section will review the psychological ID (ID) antecedents of RePlanning. It is organized around cognitive, conative, and affective ID domain (see Snow et al., 1996 for a comprehensive review). A summary of cognitive-antecedent evidence to retirement planning is presented in Table 2 below.

**Table 2:** Summary study descriptives and effect sizes for cognitive individual difference antecedents of retirement planning.

**Study**	**Antecedent**	**Outcome**	**Sample (*N*) [Age Range]**	**Design**	**ES (|*r*|)**
Hung et al., 2009	Financial literacy PT_1_ PT_2_ PT/SA_3_	Tried to save for retirement	National (1151) [18 – 108] National (566) National (1645)	X	0.46^*^ 0.42 0.52^*^
	Financial literacy PT_1_ PT_2_ PT/SA_3_	Made plan for retirement savings	National (1151) National (566) National (1645)		0.42 0.39 0.53^*^
Lusardi and Mitchell, 2007	Financial literacy(PT)	Thoughts about retirement	National (2635) [51 – 61]	X	0.12^*^
Hershey and Mowen, 2000	Perceived financial knowledge	Perceived financial preparedness	State representative adults (230) [37–88]	X	0.57^*^
Jacobs-Lawson and Hershey, 2005	Perceived financial knowledge	Retirement saving practices	National full-time workers (270) [25–45]	X	0.52^*^
Noone et al., 2010	Financial reps.	Financial preparedness	National full-time workers (1,449) [49–60]		0.32^*^
	Lifestyle reps.	Lifestyle preparedness			0.36^*^
	Psychosocial reps. Medical-health reps. Behavior-health reps.	Psychosocial preparedness Health preparedness (medical) Health preparedness (behavior)		X	0.31^*^
					0.21^*^
					0.14^*^
Heraty and McCarthy, 2015	Aging autonomy	Pension contribution	National full-time workers (8,504) [50–65]	X	0.09^*^

*PT, performance test; SA, subjective assessment; National, National-representative sample; X, cross-sectional design*.

* *p < 0.05*.

### Cognitive

#### Financial literacy

A recent, comprehensive empirical investigation of financial literacy details the conceptual and measurement definitions of the construct, as well as the extent to which it serves as a determinant of planning and saving behavior (Hung et al., 2009). Previous definitions of the construct commonly included references to specialized (financially-based) forms of declarative and procedural knowledge. Hung et al. (2009) defined financial literacy as, “knowledge of basic economic and financial concepts, as well as the ability to use that knowledge and other financial skills to manage financial resources effectively for a lifetime of financial well-being” (p.12). Operational definitions are delineated along assessment methods (objective vs. self-report) and content domains (savings, investment, debt). The Hung et al. review also indicated that objective assessments have primarily been knowledge-based. Similarly, the majority of subjective self-reports have focused on perceived knowledge as opposed to, say, financially-linked processing strategies.

Hung et al.'s (2009) empirical investigation of financial literacy's reliability and validity commenced with a retrospective cross-sectional design, using RAND's American Life Panel Data from 2006 to 2009. Four instruments purported to assess financial literacy were compared, specifically, three declarative knowledge tests and an experience-based task. Unfortunately, test-retest reliability data was available for only one of the three assessments. Construct validity was demonstrated with convergent validity between scales, with higher inter-scale correlations among the three knowledge tests (*r* range = 0.65–0.72), compared to concordance with the experimental task (*r* range = 0.33–0.35). Hung et al. (2009) also noted that other contextual factors likely impede the translation of intentions into behavior, referencing Azjen's (1991) TPB.

In a different program of research, Lusardi and Mitchell (2007) used retrospective cross-sectional data from the 2004 wave of HRS to investigate the influence of financial literacy on self-report RePlanning among employees, aged 51–61-years. The measure of RePlanning was a single anticipatory rehearsal item, “How much have you thought about RePlanning?” Only the most difficult-of-four items (compound interest) proved to be a significant predictor of thinking about retirement (*d* = 0.54).

#### Perceived financial planning knowledge

Aside from objective measures, Hershey and Mowen (2000) devised a 4-item, self-report measure of perceived financial planning knowledge. Unfortunately, a definition of perceived financial planning knowledge is not provided, but the authors generally describe the scale as domain-specific confidence, and separate from personality. In a prospective cross-sectional design among middle-age and older adults (37–88-years) Hershey and Mowen (2000) report a large effect size for the impact of perceived financial planning knowledge on the criterion, financial preparedness (*r* = 0.57). In a follow-up investigation (Hershey et al., 2003), results were replicated among a younger sample of employees (age range: 25–45 years) using a criterion of self-reported retirement saving tendencies (*r* = 0.52).

#### Retirement representations

Noone et al. (2010) conceptualize RePlanning according to a developmental process model, comprised of four stages. The first stage, *retirement representations*, is defined as, “…the strength of mental representations” (p.522). These discrepancy-based cognitions derive from prior and current knowledge in relation to desired future states. Items were generated across four planning phases (representations, timing decisions, efficacy decisions, and preparedness) and four content domains (financial, lifestyle, psychosocial, and health). However, it should be noted that student samples were used for determining preliminary-item exclusion, which is methodologically questionable in terms of item-information.

### Conative

#### Financial inhibition and activation

In a two-part study, Neukman and Hershey (2003) developed measures of financial inhibition and activation, based on Gray's (1975) physiologic-motivation model of the behavioral approach and inhibition subsystems. Adapted to the RePlanning context, Neukman and Hershey argue that envisioning retirement engenders either goal-based or fear-based motives, which are further implicated in long-term patterns of savings behavior. Items comprising the financial inhibition scale (FIS) are purported to, “…reflect a concern for negative future occurrences or apprehension associated with the financial planning and savings process,” whereas items comprising the financial activation scale (FAS) are purported to “…identify individuals who are responsive to positive future financial occurrences, or who are motivated to engage in goal-setting activities in the financial planning context” (p. 22; Neukman and Hershey, 2003).

#### Financial risk tolerance

Jacobs-Lawson and Hershey (2003, 2005) developed an attitudinal assessment of financial risk tolerance, defined as “…attitudes toward risk, specifically, as applied to financial investing for retirement” (p. 335). Conceptually, risk-tolerance is a unidimensional measure of risk-seeking, but it is assessed as a stated-preference, rather than a propensity-behavior. In a prospective cross-sectional design, Jacobs-Lawson and Hershey (2005) found that financial risk tolerance exhibits a small-medium positive effect on retirement saving tendencies (*r* = 0.16).

Working from an investment manager perspective, Grable and Joo (1997) developed a multidimensional, subjective assessment of risk tolerance. The investment manager literature most often conceptualizes risk tolerance as an attitude toward objective risk, but also allows for evaluative variation, that is, absolute-agreement on objective risk is not required for rank-ordering individuals vis-à-vis their subjective-risk preferences. As the criterion in Grable (1997) dissertation, risk tolerance was defined as, “…the maximum amount of investment risk someone was comfortable taking” (p. 13). An initial item pool (more-than 100 items) was iteratively reduced, based on empirical testing and theoretical criteria (Grable and Lytton, 1999), resulting in a 13-item instrument representing three dimensions (Investment Risk, Risk Comfort and Experience, and Speculative Risk).

#### Retirement goal clarity

Unfortunately, neither a definition nor description of scale development accompanies an initial study hypothesizing that goal clarity positively impacts retirement savings tendencies (Stawski et al., 2007). The authors describe the construct as a *process* measure of human goals (cf., Austin and Vancouver, 1996). Using a prospective cross-sectional design, results of an EFA with oblique rotation resulted in a four-factor solution with five items comprising the first factor interpreted as goal clarity and three factors interpreted as dimensions of financial planning. Items are characterized as tapping individuals' magnitude of thoughts and discussions related to retirement, as well as the setting of non-financial goals related to retirement. Test-retest reliability of goal clarity over 2 weeks was fairly strong (*r* = 0.87), considering the relatively short time-interval and fairly young-spectrum of the age distribution. Goal clarity exhibited medium positive effect sizes with a composite of RePlanning activities (*r* = 0.32) and self-reported retirement savings (*r* = 0.36).

Further concurrent validity evidence for the retirement goal clarity instrument described above and RePlanning activities was replicated in different sample (*r* = 0.41) (Hershey et al., 2007). In addition, medium—large positive effects sizes were found for goal clarity with future time perspective (FTP; *r* = 0.48), perceived financial knowledge (*r* = 0.49), and self-reported retirement savings (*r* = 0.32). A notable discrepant finding between these two studies is the positive effect of age and retirement goal clarity in the first study (*r* = 0.45) and the smaller effect in the 2007 investigation (*r* = 0.09). Finding nearly negligible age effects for middle-age working adults on non-financial aspects of retirement goals may be informative as to the developmental patterns of different goals (both in terms of *content* and *clarity*) and, by extension, the requisite planning needed to support those goals in retirement.

Much of the literature reviewed thus far has been based on non-experimental designs. However, a series of experiments conducted by Cai and Yang (2012) on decision-making under risk speaks more directly to differential effects of financial goals as a function of other factors, such as wealth, loss/gain decision domains, and goal level. In discussing the motivational factors that impact financial decisions vis-à-vis goals, Cai and Yang note that financial goal attainment (and failure) has both financial and psychological consequences. A computerized investment game consisting of repeated choices to paired certainty—risky options was used, in which expected values varied randomly within treatments, but were equal across conditions. Self-reports of general and financial risk preferences were statistically controlled in all analyses. In the first experiment, results of a mixed-factorial design, with wealth as a within-subjects factor and financial goal (vs. no goal) as a between-subjects factor, indicated that having a financial goal resulted in a small—medium positive effect on risk-aversive decisions (*r* = 0.26). In addition, within the goal treatment factor, analyses indicated a small positive effect on risk aversion just prior to goal attainment (*r* = 0.14), followed by a small - medium negative effect on risk aversion after goal attainment (*r* = −0.16). A second, mixed-factorial experiment included a “vague” and “explicit” goal condition, as well as a control condition. Results indicated a wealth effect in the “explicit” goal group, such that a small—medium positive effect size was obtained as participants neared their financial goal (*r* = 0.18), which was subsequently followed by a negative effect after goal attainment (*r* = −0.19).

#### Conscientiousness

In a prospective cross-sectional design, Hershey and Mowen (2000) utilized a three-item measure of Conscientiousness based on Mowen's (2000) 3M theory of personality. Findings indicated that Conscientiousness has small—medium positive effect sizes in relation to various composite retirement variables, including, retirement relevance (*r* = 0.13), self-reported financial preparedness (*r* = 0.25), and perceived financial knowledge (*r* = 0.32).

#### Proactivity

Griffin et al.'s (2012a) recent work in the RePlanning domain makes two notable contributions to the field: First, the authors strongly advocate for the use of grounded theory in studies of RePlanning antecedents and outcomes. In particular, they noted that Azjen's (1991) Theory of Planned Behavior was absent in extant research of RePlanning (Griffin et al., 2012a). Second, the authors stress the need to incorporate temporal factors into studies of RePlanning, either through longitudinal designs or latent-temporal construct measurement, such as FTP or time-discounting. In a prospective cross-sectional design, Griffin et al. (2012b) assessed trait proactivity with a six-item scale adopted from Bateman and Crant's (1993) proactive personality scale. Proactive persons are described as forward-thinking self-starters who persevere in behaviors aimed at changing their environment for the better. Results indicated that proactivity had a medium positive effect size with a composite behavioral measure of RePlanning activities (*r* = 0.26). A summary of antecedent evidence to retirement planning, by “conative” and “affective” domains is presented in Table 3 below.

**Table 3:** Summary study descriptives and effect sizes for conative and affective individual difference antecedents of retirement planning.

**Conative antecedents**					
**Study**	**Antecedent**	**Outcome**	**Sample (*****N*****) [Age Range]**	**Design**	**ES (*****r*****)**
Grable and Lytton, 1999	Financial risk toler.	Acceptable investment-risk	University faculty and staff (1,075) [22–77]	X	0.54*
Hershey and Mowen, 2000	Conscientiousness FTP	Financial preparedness	US state-rep. adults (230) [37–88]	X	0.25* 0.58*
Grable and Lytton, 2003	Finance-risk toler.	% of equities in portf. % of fixed-income in portf.	National sample (303) [22–61]	X	0.31* 0.32*
Neukman and Hershey, 2003	Planning drive Planning worry	Past year's ret. savings Past year's ret. savings	US national rep workers (270) [25–45]	X	0.32* 0.25*
Jacobs-Lawson and Hershey, 2005	Finance-risk toler. FTP	Retirement saving acts	US full-time employees (265) [25–45]	X	0.16* 0.26*
Cai and Yang, 2012	Finance goal clarity	Risk-aversion	Undergraduate students (95) [18–24]	E	0.26*
Hershey et al., 2007	Ret. goal clarity	Planning activities	Full-time employees (265) [25–45]	X	0.41*
Stawski et al., 2007	Ret. goal clarity Ret. goal clarity	Past year's ret. savings Planning activities	Convenience sample (100) [19 – 63]	X	0.64* 0.41*
Noone et al., 2010	FTP FTP FTP FTP	Financial preparedness Health preparedness Lifestyle preparedness Psychosocial preparedness	National full-time workers (1,449) [49–60]	X	0.16* 0.15* 0.37* 0.10*
Griffin et al., 2012b	Proactivity	RePlanning activities	Full-time employees (432) [45–70]	X	0.33*
Hurd et al., 2012	Conscientiousness Agreeableness	Financial prep. for ret.	US national sample (7,500) [66–69]	X	0.14*/0.03 −0.03/−0.10
Muratore and Earl, 2010	Mastery	Public protection Self-insurance Self-protection	Australian full-time retirees (549) [48–78]	X	0.02 0.20* 0.23*
Kerry and Embretson, 2017	Conscientiousness Agreeableness	RePlanning	Full-time employees (254) [50–65]	E	0.11/0.29* 0.15/0.06
**Affective antecedents**					
**Study**	**Antecedent**	**Outcome**	**Sample (*****N*****) [Age Range]**	**Design**	**ES (*****r*****)**
Hershey and Mowen, 2000	Emotional Stability	Financial preparedness	State-representative adults (230) [37–88]	X	0.16*
Petkoska and Earl, 2009	FTP (Fatalistic) FTP (Fatalistic) FTP (Hedonistic) FTP (Past-Positive)	Finance/general plan. Interpers./leisure plan. Interpers./leisure plan. Finance/general plan.	Australian financial institute employees (377) [50–66]	X	0.19* 0.16* 0.22* 0.15*
Hurd et al., 2012	Neuroticism Extroversion Openness	Financial preparation for ret.	US national sample (7,500) [66–69]	L_3−year_	−0.10/−0.10 −0.05/−0.13 0.01/0.09
Heraty and McCarthy, 2015	Sporadic age-salience Negative reactivity	Pension contribution Pension contribution	National full-time workers (8.504) [50–65]	X	−0.12*−0.09*
Kerry and Embretson, 2017	Neuroticism Extroversion Openness	RePlanning	Full-time employees (254) [50 – 65]	E	−0.13/−0.31* 0.36*/0.39* 0.31*/0.35*

Entries listed in chronological-publication order. X, cross-sectional, L, longitudinal. Portf, portfolio; Prep, preparedness. Plan, planning; Interpers, interpersonal.^*^p < 0.05.

### Affective

#### Emotional stability

Hershey and Mowen (2000) evaluated the impact of a four-item measure of emotional stability on RePlanning and its antecedents. While structural coefficients in the latent model indicated positive effects of emotional stability on FTP, as an antecedent to RePlanning, evaluation of the correlation matrix indicated small – medium *negative* effects on perceived financial knowledge, (*r* = −0.28), and financial preparedness (*r* = −0.14).

#### FTP

In the same study cited immediately above, Hershey and Mowen (2000) posited FTP to be a central trait in their hierarchical framework of personality. That is, they believed that one's orientation to time would mediate cardinal (i.e., elemental) traits and various RePlanning antecedents. A locally developed four-item measure indicated large positive effect sizes with perceived financial knowledge (*r* = 0.48) and perceived financial preparedness (*r* = 0.50). Concurrent validity for the instrument was extended in a prospective cross-sectional design, where FTP was found to positively predict a five-item composite measure of retirement savings (*r* = 0.26) (Jacobs-Lawson and Hershey, 2005).

In a similar vein, Petkoska and Earl (2009) examined orientation to time in relation to RePlanning for four different domains: finances, health, interpersonal/leisure planning, and work. Unique to this study, the authors employed Zimbardo and Boyd (1999) five-dimension inventory of time perspective (future, present hedonistic, present fatalistic, past positive, and past negative). Using a prospective cross-sectional design, results indicated small-medium effect sizes across time perspective dimensions and planning domains. Specifically, the present fatalistic dimension negatively related to financial planning for RePlanning (*r* = −0.18). Contrary to expectations, however, the past positive dimension was positively related to financial RePlanning (*r* = 0.16). This may conceptually overlap with Mael and Ashforth's (1992) *sentimentality* construct, defined as, “the tendency to retain emotional or tangible ties to one's past.” (p.122). For the interpersonal/leisure planning domain, both the present fatalistic and present hedonistic dimensions exhibited small effects, but in opposite directions, (*r* = −0.15 and 0.21, respectively). Taken together, we consolidate propositions delineated by the Five-Factor personality model for examining mechanisms and outcomes of RePlanning, displayed in Table 4 below.

**Table 4:** Summary propositions for five-factor personality effects on well-being in retirement.

**Big-five factor**	**Descriptive proposition**
Neuroticism	Neuroticism will negatively relate to well-being in retirement. Perceived-voluntariness of the R.D. will weaken the effect.
Extraversion	Extraversion will positively relate to well-being in retirement. Perceived-voluntariness of the R.D. will strengthen the effect.
Openness	Openness will positively relate to well-being in retirement. Perceived-voluntariness of the R.D. will weaken the effect.
Agreeableness	Agreeableness will positively relate to well-being in retirement. Perceived-voluntariness of the R.D. will strengthen the effect.
Conscientious	Conscientiousness will positively relate to well-being in retirement. Perceived-voluntariness will R.D. the positive effect.

*Researchers interested in more narrow (specific) or general (broad) personality constructs related to retirement planning and outcomes should consult research from Colin G. DeYoung. R.D., retirement decision*.

### Summary of psychological antecedents of retirement planning

In summary, multiple psychological IDs variables in the cognitive, conative, and affective domains have received empirical support as antecedents of RePlanning. However, the strength of evidence regarding the predictive validity of constructs varies across these three intrapsychic domains.

As a whole, the ideographic approach to the study of RePlanning, particularly in the cognitive domain, could be greatly advanced by the use of longitudinal designs to evaluate the effects of early-declarative investment knowledge on cumulative saving strategies and retirement plans over time (Pressley et al., 1989). For the conative and affective domains, replication and expansion of focal constructs would seem to be a crucial objective.

## General discussion

We began this review by illustrating the complementarity of phased-retirement and career-stage models. Consequently, we extracted that the boundary condition to decision-making paradigms of retirement research (voluntariness) is generalizable to newer paradigms emphasizing transition-processes and career development. That is, factors preceding the retirement decision can reasonably be expected to impact transition-adjustment processes and career development prospects, as well. This served to broaden retirement planning's scope as a bidirectional lens for understanding normative changes to the changing nature of work and retirement.

After chronicling RePlanning's process-modals (in-/formal) and content-domains (non-/financial), we reviewed the empirical research on its antecedents. We concluded that relatively more programmatic research has been conducted on cognitive, compared to conative and affective antecedents of RePlanning. The remainder of our Discussion will elaborate on the conceptions and empirical evidence for RePlanning. This is guided by two thematic research questions derived from our observations of the empirical literature: (1) does planning uniformly lead to better outcomes and, by extension, can we identify the conditions under which different forms of planning will exhibit stronger, weaker, or unanticipated effects? and (2) can alternative-mechanisms be identified that may account for observed-effects of planning? We conclude by articulating current limitations and synthesis for future research.

### Conceptions of retirement planning

Our review of the process-modals and content-domains of RePlanning indicated a conceptual conflation of informal and non-financial planning behavior. On the one hand, RePlanning propensities (process-modals) are partially determined by institutional variations in entitlement program designs. On the other hand, recent research has indicated cross-domain consistency for information-seeking propensities in a large sample of older adults (*r* = 0.17; Carr, 2015). In terms of domain-specificity, there is also consistent findings for the relatively robust-effects of non-financial education.

### Antecedents of retirement planning

Our empirical review of the antecedents of RePlanning indicated voluminous and consistent effects for claimed-, declarative-, and procedural-knowledge of retirement planning on subsequent behaviors and wealth indicators. Indeed, most studies in the cognitive domain have been conducted with respect to financial forms of RePlanning. The empirical evidence for affective- and conative- antecedents was weaker, both in terms of design-strength and systemization of observed effects. In particular, financial-risk tolerance and future time perspective are two constructs of substantive relevance to the RePlanning domain, and we recommend their priority in future research. Importantly, recent research has indicated that personal resilience was greater-associated with retiree well-being compared to socioeconomic satisfaction (Nalin and França, 2015). Toward a firmer foundation of conative and affective antecedents' research, we tentatively offered descriptive-propositions based on the five-factor model of personality in Table 4. Again, interactions with perceived-voluntariness of the retirement decisions are included for more informative examination pertaining to antecedent-outcome gaps.

### Limitations of retirement planning research

Many limitations to the empirical research on RePlanning abide earlier observations, such as overuse of non-experimental, particularly, cross-sectional designs, as well as over-reliance on self-report instruments (Feldman, 1994). Comparatively, more progress has been made on increasing longitudinal examinations of retirement plans, whereas inadequately narrow assessment methods persist. The implications of this mono-method bias are not, yet, fully understood. Recent meta-analytic evidence, however, suggests that hetero-methods implicate relevant constructs for, both organizational and retirement scholars (e.g., *impulsivity;* Sharma et al., 2013).

RePlanning, *ceteris paribus*, accelerates intentional workforce exit (*r* = −0.21; Kerry and Embretson, 2017). Substantively, however, less retirement planning should not be interpreted as indifference to continued labor force participation. Extrapolating, chronological age has exhibited comparatively weaker correlates with retirement planning behaviors than, both self-reported retirement proximity and subjective life expectancy (SLE). These findings may, indirectly, reflect the common omission of withdrawal-intentions (or perceived-voluntariness) in retirement planning assessments. Ancillary evidence for this supposition comes from findings of positive correlates between retirement attitudes and planning-process involvement (Kalokerinos et al., 2015a).

We echo the imperative of interdisciplinary approaches for examining the antecedents and outcomes of RePlanning. The most well-designed, parochial programs of research will likely be of little use. A sterling example (and substantive miss) is the case of the Thaler and Benartzi (2004) “Save More Tomorrow,” designed to combat hyperbolic-discount through deferred, auto-escalated retirement contributions. Unfortunately, the wage-stagnation from exponential growth in healthcare-expenditures effectively neutralized the carefully-designed intervention. More general, normative-solutions to retirement planning (e.g., savings-commitment devices) are necessarily subject to individual-heterogeneity of preferences and motivations.

Related, a particularly understudied area deserving future empirical research is the functional links between wealth, health, planning, and retirement. While the wealth-health link has historically and, phenomenologically, presented challenging, advanced-quantitative methods and voluminous public-access data should invite more sophisticated analyses with the goal of deeper understanding. We concur with Taylor and Schaffer's recommendation, that “The pervasiveness of this factor…suggests that it should be incorporated into any model of planning” (p. 253). Only cursory attention has been paid, unfortunately. Based on the most-current MA data on the health-wealth link and RePlanning, Topa et al. (2012) concede that, “a clear understanding of the health-retirement relationship has not been provided” (p.137). We are compelled to conclude that the health domain is the opportune, though currently neglected, area of study for retirement researchers.

In principle, though not strictly limited to retirement planning research, we are obliged to recommend the abandonment of “push-pull” terminology. At greatest issue, perhaps, is the implicit relegation of older workers as “passive” agents through retirement, which is antithetical to more sophisticated paradigms of “active aging.” Standalone, “push-pull” factors have served only atheoretical, descriptive heuristics for organizing motivations toward retirement. Specifically, the “push-pull” factors are ascribed negative-positive valences, respectively for work-retirement transitions (Shultz et al., 1998). Its primary limitation, however, is the omission of directional-referent or origin (e.g., work → turnover/retirement, turnover/retirement → reentry). As Williamson et al. (1992) exemplify, “The negatives about your work and the positives of retirement are the “rewards”…these can be directly compared to the positives that will be missed and possible negatives…which together are the “costs” of retirement. (*p*. 35). Shultz et al. (1998) similarly note that, “the same event may be rated as either a push or a pull by different workers” (p.46). Withstanding the generality of push-pull labels, we consider potential leveraging of their constraints for more actionable-research toward understanding organizational phenomena. We elaborate on the prospective retention of “push-pull” factors in a later table of plausible-rival hypotheses (see Table 5).

**Table 5:** Summary-plausible rival explanatory propositions and provocations for advancing future empirical research on retirement planning.

**Testable proposition**	**Supplementary rationale**
Planning-as-preparation vs. planning-as-expectancy (*financial resourcefulness*)	Generally, planning exhibits most-pronounced, positive effects in early retirement. Contrary, job-status is associated with most-pronounced, negative effects in early retirement. A confound to job-status, however, is the increased financial resources for retirement, *ceteris paribus*. If planning serves a preparatory-buffer function, then, positive effects should be robust to perceived-voluntariness of retirement decision (retrospective study). In this case, job-status may positively interact with planning to strengthen the positive effect. On the other hand, if planning serves a nonwork- expectancy function, then job-level should reduce the positive-effect, again, controlling for perceived-voluntariness of the retirement decision.
Career continuity vs. vocational adaptability (*non-finance resourcefulness*)	Increased emphasis on job-crafting and psychological contracts posit the attraction of flexible-work arrangements to employees. Contrary, cultivation of hobbies and interests, as well as unemployment exposure, may furnish nonwork-coping mechanisms from cumulative off-job experience. Taken together, future research may examine the cumulative effects of continuity-affording financial resources versus the discontinuity-affording vocational engagement.
Age-related changes in FTP vs. goal-related changes in FTP	Workforce aging can be viewed *relatively* (as cohort-effect) or *absolutely* (as longer life expectancies). Additionally, lifespan researchers have derived opposing predictions for age-related changes in FTP, originating from complements of the lifecycle model. That is, retirement ‘process planning' theories were predicated on early-childhood development paradigms, whereas organizational theories derived predictions from gerontological paradigms.
Self-regulatory focus as-linkage across workplace and non-workplace domains^2^	What is the relative-impact of work – nonwork valuation (affect) and short – long time horizons (cognitive) as common causes to work and retirement? Evidence from a recent, randomized-controlled trial (RCT) of subjective life expectancies (SLEs) was mixed as-to FTP generally, but a follow-up RCT with occupational-FTP is currently underway (Kerry and Embretson, 2017). An extension to occupational-adaptations of the FTP instrument (O-FTP) is also relevant. Specifically, it has been argued that customization of job characteristics of greater value to older workers, retention may increase as a function of extending one's O-FTP. A plausible rival hypothesis to observed-retention from enhancing job-characteristics may be that the psychological costs of *delaying* retirement are reduced. This alternative hypothesis can be fairly easily evaluated in an experimental paradigm by factorially crossing self-report retirement planning, intended retirement, and O-FTP with two version of a delay discounting task: One based on descriptive info (hypothetical choice), and one based on experiential info (deferral of gratification).
Extend DM-paradigms of descriptive assessments to experiential assessments	Hadar and Fox (2009) elaborate on the information-asymmetries between descriptive and experiential decision-making. Computationally, we've known for some time that the coefficient of relative-risk aversion is, reciprocally, the elasticity of intertemporal substitution (Kocherlakota, 1990). Expositorily, Posner (1995) observes, “A discount rate and an interest rate are the same…the rate at which present and future costs or benefits are made equivalent.” (p. 92). Recent empirical evidence implicates the substantive impact of these tenets from the DM paradigm. For example, Mata et al. (2011) provide meta-analytic evidence for reverse-findings of age-related differences in risky choice, specifically, as a function of descriptive- or experiential-DM assessment paradigms. Similarly, Paglieri (2013) reported the scalar differences between delay-discounting assessed from real-time behavioral-observations (preserving waiting costs) versus descriptive-reports (stated postponements). These temporo-methodological phenomena have received scant empirical attention from extant psychological studies of retirement planning, c.f., (Bidewell et al., 2006).
Reorient “push-pull” factors, from retiree outcomes, to examine various organization phenomena	An example of “push-pull” redirect to organization practices, could be the consideration of antipodes (+/-) to well-studied phenomena. For example, in Ng and Feldman (2007) developmental distinction between organizational- and occupational embeddedness, they observed, “Like mobility, then, embeddedness has its own set of potential negative consequences for organizations.” (p.347). Extrapolating, the recent recession may be conjectured as a pull factor for extending employment (though negative-valence), while health reform aimed at broadening individual-coverage and markets may be could be construed as a push factor (though positive-valence).

In summary, existing RePlanning research has identified relevant correlates and broadened the scope of factors to consider that may affect retiree WELL-BEING, however, it has inadequately addressed these implications for future employees. This is critical, because *some* of the work-design principles to accommodate the relative-aging workforce will abide technologic shifts, as well as absolute-aging more generally in terms of longer life expectancies. Before concluding, in the next section, we aim to synthesize findings from RePlanning research toward future-explanatory propositions.

### Synthesis and testable propositions for future research

In agreement with Griffin et al. (2012a), results of the current review suggest that future research on the antecedents of RePlanning may be best-served by incorporating time-related constructs, be it subjective life expectancies, planning horizons, or FTPs. One particularly challenging issue in the psychological research of RePlanning has been the conflation of current valuation with longevity perspectives. As Posner succinctly summarizes, “A discount rate and an interest rate are the same…the rate at which present and future costs or benefits are made equivalent.” (p. 92). Toward future integration, we offer Table 5 below for consolidated, though non-exhaustive, explanatory-propositions for antecedents and outcomes of retirement planning. It is the objective to provoke and stimulate more thoughtful empirical questions toward integrating observed-planning phenomena across work—retirement scholarship domains. Propositions are primarily pitted at the intersection of demography (global aging) and labor modularization. The premises span both substantive theory and methodological technique.

## Conclusion

Recent empirical evidence supports the potentially meaningful separation of domain-valuation and work-related time perspective (Tschopp et al., 2015). Parsing these factors is as an important area for future research. Similarly to antecedents, research on outcomes of RePlanning may contribute most at two “temporal extremes”: The first is the correlates of RePlanning to other work-related variables (e.g., embeddedness, commitment), and the second is on the distal-effects of RePlanning in later-retirement (e.g., it has been speculated that the effects of planning may subside after adjustment).

The increasing overlap of work and retirement contexts is concomitant with continuously aged and mobile workforces, and both compels precise application of lifespan motivation theories (Abraham and Hansson, 1995). In terms of timescales, age effects may be attributable to, both the personnel and the institutions they occupy. For example, the ostensible age-effect of industry may be work-redesign for Baby Boomers (cohort-scale), but this necessarily implicates discontinuity of the information age (period-scale). Pivoting to persons occupying the workforce, the “greying” workforce is, perhaps, a mischaracterization inasmuch life expectancies persist. For retirement planning, the difference is profound, because some of the “temporally” relevant lessons of the currently-aging cohort can be expected to abide future technologic shifts (Schaie, 1965).

## Author contributions

The author confirms being the sole contributor of this work and approved it for publication.

### Conflict of interest statement

The author declares that the research was conducted in the absence of any commercial or financial relationships that could be construed as a potential conflict of interest.

## Footnotes

^1^We concur, but note that the commonly cited boundary condition to the DM paradigm, voluntariness, is symptomatic of a larger, endogeneity error (explanatory limit) in psychological retirement models. For example, the exogenous factors impacting retirement DM, such as objective health or macroeconomic conditions, necessarily affects “adjustment” and “career development” post-retirement, as well.

^2^The Boolean search-term protocol used to identify the empirical literature is provided in online-Appendix B.
