# Supplementary material for: Psychological Antecedents of Retirement Planning: A Systematic Review
Source: Front Psychol. 2018 Oct 9;9:1870. doi: 10.3389/fpsyg.2018.01870 (PMC6189550; doi:10.3389/fpsyg.2018.01870)
Supplement: Supplementary file 1 [file Data_Sheet_1.pdf]

APPENDIX A – CONCEPTS OF RETIREMENT PLANNING

Table APPENDIX A

Summary Labels, Definitions, and Measurement Characteristics of Retirement Planning Identified from the Psychological Literature

Label	Definition or Description	Source	Measurement Descriptives	Sample Item
retirement plans	"…behavioral intentions about ret." (p.140)	Ekerdt, DeViney, & Kosloski (1996)	Single-item self-report Dummy-coded	complete, partial, or never ret.; serial-transition, uncertain
financial preparedness	"…an understanding of how much money would be needed to adequately meet ret. expenses and whether the appropriate computations had been made to ensure one would be financially solvent during postemployment period" (p.691)	Hershey & Mowen (2000)	4-item self-report 7-point Likert-type (1) Strongly disagree – (7) Strongly agree	I know how much money I (we) will need to comfortably retire.
retirement planning	"…a cluster of loosely related cognitions and behaviors that are partially substitutable and individually represent the construct to some unknown and varying degree…at simplest level…can be represented as an intention to the form ret. will take…most direct measure is anticipatory rehearsal. That is, to what extent are workers thinking about ret. and discussing it with their friends and coworkers…such activities serve as an anchor by which to evaluate and add meaning to the other measures" (p.162)	Kosloski, Ekerdt, & DeViney (2001)	Mixed-format:<br>*Intention*: Single dummy-coded variable.<br><br>*Subjective probability*: 2-items, from 1 (no chance) to 10 (certainty).<br><br>*Anticipatory Rehearsal*: 2-item self-report 4-point Likert-type (a lot) – (hardly at all)	stop work altogether, work fewer hours, change kind of work, never stop working, no plans<br><br>Chances you'll be working full-time after reaching age 62 / 65<br><br>How much have you thought about ret. / discussed ret. with your friends or coworkers?

retirement anticipation and preparation	Multifaceted: "Anticipation and preparation for ret. variables included proximity to ret., ret. fund calculation, ret. savings amount, confidence in government program, and workplace financial education and advice" (p.81).	Kim, Kwon, & Anderson (2005)	Mixed-format: *Proximity*: difference between age and expected ret. *Ret. Fund Calculation*: Y/N *Savings Amount:* dummy-coded with prescribed ranges *Confidence in government program*: 2-item self-report 4-point Likert-type (Not at all confident) – (Very confident). *Workplace financial education and advice:* Y/N	In the past 12 months, has an employer or work-related retirement plan provider given you educational material, information, or seminars about retirement planning and savings?
retirement planning	"Having thought about ret." (p.216).	Lusardi & Mitchell (2007)	Single-item self-report; 4-point Likert-type (A lot) – (Hardly at all)	How much have you thought about RePlanning?
retirement planning activity level	"…the frequency of both information seeking and instrumental planning activities that had occurred in the past 12 months" (p.31).	Hershey, Jacobs-Lawson, McArdle, & Hamagami (2007)	10-item self-report; 7-point Likert-type (Strongly disagree) - (Strongly agree)	Frequently read articles/brochures on investing or financial planning.
financial planning activities	"…span a wide range of behaviors. One class of behaviors involves information-seeking activities" and "…instrumental behaviors".	Stawski, Hershey, Jacobs-Lawson (2007)	9-item self-report; 7-point Likert-type (Strongly Disagree) – (Strongly Agree)	Assessed your net worth.
retirement plans	"at what age they planned to permanently stop working with the organization"	Luchak, Pohler, & Gellatly (2008)	Open-item, self-report	At what age do you plan to stop work completely from your current organization?
retirement planning	Multi-domain goals and behaviors, "in preparation for retirement" (p.249)	Petkoska & Earl (2009)	36-item self-report Y/N	Made new friends recently

Retirement planning tendencies	"active involvement in investment, regular saving, reviewing investment performance, using several sources of investment info"	Hira, Rock, & Loibl (2009	8 behavioral variables analyzed separately.<br>6 on 5-point Likert-type scale (1) Strongly disagree - (5) Strongly agree. 1 on 5-point Likert-type scale (1) Hardly - (5) Often. 1 Y/N	Review and compare investment performance
Retirement preparation	"...effort invested by individuals, while still employed, to provide for their well-being in ret." (p.99).	Muratore & Earl (2010)	28-item self-report; 5-point Likert-type (Very small amount of effort) – (Very large amount of effort)	*Public Protection* - Applying for a seniors concession allowance<br>*Self-insurance* - Checking your superannuation fund's performance<br>*Self-protection* – Participating in one or more leisure planning or wellbeing workshops, seminars, or courses on ret. which were not government-run
Retirement planning[b]	"…goal-directed thoughts and behaviors that promote good health and provide financial security, fulfilling lifestyles, and rewarding roles in ret." (p.522)	Noone, Stephens, & Alpass (2010)	2-6-item self-report[c] 5-point Likert-type (Definitely not true of me) – (Definitely true of me)	It is likely that I will own a home w/o a mortgage in ret.
Retirement proactivity[a]	"general proactive planning for ret.…such as active exploration, planning, and monitoring" (p.108)	Griffin, Loe, & Hesketh (2012)	11-item self-report; 5-point Likert-type (Not at all) – (A great deal)	Develop knowledge and skills to help you cope well with ret.
Retirement preparation	"…the extent to which participants were engaged in preparing for their ret., including financial and non-financial planning" (p.132).	Griffin, Loe, & Hesketh (2012)	15-item self-report; 5-point Likert-type (Not at all) – (A great deal)	Actively seeking professional financial advice

Notes. [a] items adapted from existing measure, [b] item-length varies across planning domains. Ret. = retire/ment. RePlanning = retirement planning, RePrep = retirement preparation. Entries listed in publication-chronological order.

APPENDIX B – SYSTEMATIC-KEYWORD SEARCH PROTOCOL

Summary Boolean Search-Term Protocol for Identifying and Extracting Empirical Literature

Database	Boolean Search Terms (in **title** or **abstract**)	1986 - 1995	1996–2005	2006-2016
ProQuest PsycARTICLES & Dissertations	"Retire*" AND (plan* OR prep* OR saving*) AND (antecedent* OR determinant* OR cause* OR predictor*)	150	135	188
Medline	"Retire*" AND (plan* OR prep* OR saving*) AND (antecedent* OR determinant* OR cause* OR predictor*)	14	52	99
JSTOR	"Retire*" AND (plan* OR prep* OR saving*) AND (antecedent* OR determinant* OR cause* OR predictor*)	12	15	9
ERIC	"Retire*" AND (plan* OR prep* OR saving*) AND (antecedent* OR determinant* OR cause* OR predictor*)	7	8	21

Database	Boolean Search Terms (in **title** or **abstract**)	1986–1995	1996–2005	2006-2016
ProQuest PsycARTICLES & Dissertations	"Retire*" AND (plan* OR prep* OR saving*) AND (outcome* OR result* OR consequence* OR effect*)	150	236	349
Medline	"Retire*" AND (plan* OR prep* OR saving*) AND (outcome* OR result* OR consequence* OR effect*)	41	85	222
JSTOR	"Retire*" AND (plan* OR prep* OR saving*) AND (outcome* OR result* OR consequen* OR effect*)	18	36	26
ERIC	"Retire*" AND (plan* OR prep* OR saving*) AND (outcome* OR result* OR consequence* OR effect*)	8	9	49

APPENDIX C – DEMOGRAPHIC ATENCEDENTS OF RETIREMENT PLANNING

APPENDIX C

Summary Study Descriptives and Effect Sizes for Demographic Antecedents of Retirement Planning

Study	Antecedent	Outcome	Sample	Design	ES (|*r*|)
Lusardi et al. (2001, 2006, 2007)	Wealth	retirement planning	HRS; Rand ALP	X	(.07, .37)
Topa et al. (2009)	Wealth	retirement planning	Multisource	MA	.29[a]; .37[b]
Dwyer & Mitchell (1999)	Health	expected retirement	HRS	X	.15
Benitez-Silva and Dwyer (2002)	Health	thoughts of retirement	HRS	L	.12
Topa et al. (2009)	Health	retirement planning	Primary studies	MA	.05
EBRI (2015)	Gender	formal retirement plan	US census data	X	.24
Montalto, Yuh, and Hanna (2000)	Race	expected retirement	Survey of Consumer Finances	X	.21
Stawski et al (2007)	Age	planning behavior	US public convenience sample	X	.32
		retirement savings		X	.26

Note. Values in parentheses indicate range of effect sizes across primary studies. X = cross-sectional, L = longitudinal, MA = meta-analysis. HRS = U.S. Health and Retirement Study, ALP = American Life Panel. EBRI = Employee Benefits Research Institute. [a]Objective indicators, [b]Subjectve indicators.

APPENDIX D – SITUATIONAL ANTECEDENTS OF RETIREMENT PLANNING

APPENDIX D

Summary Study Descriptives and Effect Sizes for Situational Antecedents of Retirement Planning by Job Characteristic and Interpersonal Domains

Job Characteristic					
Study	Antecedent	Outcome	Sample (*N*) [Age Range]	Design	ES *r*
Kosloski et al (2001)	Intrinsic Job Reward	Retirement planning composite	US national rep (*3,936*) [51 – 62]	X	.21
	Social Relations at Work	Retirement planning composite	US national rep (*3,936*) [51 – 62]	X	.18
	Ascendance Opportunity	Retirement planning composite	US national rep (*3,396*) [51 – 62]	X	.08
Taylor & Shore (1995)	Org-filled retirement needs	Retirement financial concerns	US Firm employees (*303*) [19 – 71]	X	.27
	Org-filled retirement needs	Social retirement expectations	US Firm employees (*303*) [19 – 71]	X	.31
	Organization commitment	Planned retirement age	US Firm employees (*303*) [19 – 71]	X	.22
	Organization commitment	Retirement financial concerns	US Firm employees (*303*) [19 – 71]	X	.32

	Organization commitment	Retirement social expectations	US Firm employees (*303*) [19 – 71]	X	.36
	Organization commitment	Retirement leisure expectations	US Firm employees (*303*) [19 – 71]	X	.12
	Job satisfaction	Planned retirement age	US Firm employees (*303*) [19 – 71]	X	.20
	Job satisfaction	Retirement financial concerns	US Firm employees (*303*) [19 – 71]	X	.28
	Job satisfaction	Expected retirement adjustment	US Firm employees (*303*) [19 – 71]	X	.04
	Job satisfaction	Retirement social expectations	US Firm employees (*303*) [19 – 71]	X	.42
	Pay satisfaction	Planned retirement age	US Firm employees (*303*) [19 – 71]	X	.01
	Pay satisfaction	Retirement financial concerns	US Firm employees (*303*) [19 – 71]	X	.22
	Pay satisfaction	Retirement social expectations	US firm employees (*303*) [19 – 71]	X	.31
	Pay satisfaction	Retirement leisure expectations	US firm employees (*303*) [19 – 71]	X	.12
van Dam et al (2009)	Future manageable workloads	Early retirement intentions	Dutch full-time employees	X	.18

			(*346*) [50-65]		
	Future quality work tasks	Early retirement intentions	Dutch full-time employees (*346*) [50-65]	X	.44
Topa et al. (2009)	Negative work conditions	Retirement planning	K = 2 (*1038*)	MA	.15
	Work involvement	Retirement planning	K = 4 (*4,451*)	MA	.31
	Job satisfaction	Retirement planning	K = 6 (*2,216*)	MA	.34

Interpersonal

Study	Antecedent	Outcome	Sample (*N*) [Age Range]	Design	ES *r*
Ekerdt et al (1996)	Married	Plans for retirement	US national rep (*5,072*) [51-61]	X	.13
Kosloski et al. (2001)	Married	Earlier planned retirement	US national rep (*4,147*) [51 – 62]	X	.16
	Married	Thoughts about retirement	US national rep (*4,147*) [51 – 62]	X	.18
Elders & Rudolph (1999)	Married	Attended a retirement seminar	US retirees (1,781) [51 – 62]	X	.25
vanRooij et al (2012)	Married	Retirement plan adherence	Dutch heads of households (*1,091*) [22-90]	X	.05
Lusardi (2003)	Observe worse financial status of older sibling	Thoughts about retirement	US national rep (*4,147*) [51 -61]	X	.12
	Observe parents in nursing home	Thoughts about retirement	US national rep (*4,147*) [51 -61]	X	.17

	Observe parents with terminal illness	Thoughts about retirement	US national rep<br>(*4,147*) [51 -61]	X	.16
Chalmers et al (2008)	Average proximity of peer retirement	Decision to retire	Oregon public sector employees (*71,923*) [58 – 62]	L	.31
Duflo & Saez (2002)	Average within-department participation	Employer-sponsored retirement plan participation	Public university staff and faculty (*11,360*) [20 – 64]	X	.31
Duflo & Saez (2003)	Monetary incentive for attending benefits fair (1-F)	Retirement plan enrollment	Public university staff<br>(*6,200*) [20-59]	RCT<br>2-Factor<br>(nested)	.19
	Monetary incentive for attending benefits fair (2-F)	Retirement plan enrollment	Public university staff<br>(*6,200*) [20-59]	RCT<br>2-Factor<br>(nested)	.12

Note. Entries listed in chronological order. X=cross-sectional design, RCT = Randomized Control Trial, MA=meta-analysis.
